# Mitochondria in the biology, pathogenesis, and treatment of hepatitis virus infections

**DOI:** 10.1002/rmv.2075

**Published:** 2019-07-19

**Authors:** Changbo Qu, Shaoshi Zhang, Yang Li, Yijin Wang, Maikel P. Peppelenbosch, Qiuwei Pan

**Affiliations:** ^1^ TEDA Institute of Biological Sciences and Biotechnology Nankai University Tianjin China; ^2^ The Key Laboratory of Molecular Microbiology and Technology Ministry of Education Tianjin China; ^3^ Department of Gastroenterology and Hepatology Erasmus MC‐University Medical Center Rotterdam The Netherlands; ^4^ Department of Pathology and Hepatology Beijing 302 Hospital Beijing China

**Keywords:** hepatitis virus, mitochondria, pathogenesis, treatment

## Abstract

Hepatitis virus infections affect a large proportion of the global population. The host responds rapidly to viral infection by orchestrating a variety of cellular machineries, in particular, the mitochondrial compartment. Mitochondria actively regulate viral infections through modulation of the cellular innate immunity and reprogramming of metabolism. In turn, hepatitis viruses are able to modulate the morphodynamics and functions of mitochondria, but the mode of actions are distinct with respect to different types of hepatitis viruses. The resulting mutual interactions between viruses and mitochondria partially explain the clinical presentation of viral hepatitis, influence the response to antiviral treatment, and offer rational avenues for novel therapy. In this review, we aim to consider in depth the multifaceted interactions of mitochondria with hepatitis virus infections and emphasize the implications for understanding pathogenesis and advancing therapeutic development.

**Abbreviations**
HCC: hepatocellular carcinoma
MAVS: mitochondria antiviral‐signaling protein
mtDNA: mitochondrial DNA
TLR9: Toll‐like receptor 9
STING: stimulator of interferon genes
AICAR: 5‐aminoimidazole‐4‐carboxamide ribonucleotide
HBx: HBV X gene product
ETC: electron transport chain
PAMP: pathogen‐associated molecular patterns
ORF2: open reading frame
Mfn1: mitofusin‐1
Mfn2: mitofusin‐2
Opa1: optic atrophy 1
Drp1: dynamin‐related protein 1
RLR: RIG‐I‐like receptor
ROS: reactive oxygen species
MPTP: mitochondrial permeability transition pore
VDAC: voltage‐dependent anion channel
ANT: adenine nucleotide translocator
CypD: cyclophilin D
CsA: cyclosporine A

## 1 INTRODUCTION

Hepatitis or liver inflammation is one of the most common liver diseases that imposes a heavy global health burden.^1^, ^2^ Acute hepatitis is either self‐resolving or develops into chronic hepatitis and subsequently progresses to cirrhosis or hepatocellular carcinoma (HCC).^3^ The main etiologies include infection, metabolism, and autoimmune‐related causes. Viral infections including hepatitis A, B, C, D, and E viruses (HAV, HBV, HCV, HDV, and HEV, respectively) are the leading causes (Table 1).

**Table 1:** Features of hepatitis virus infections

	HAV	HBV	HCV	HDV	HEV
Size, nm	27‐32	42	55‐62	36‐43	27‐34
Genome	+ssRNA	Partially dsDNA	+ssRNA	−ssRNA	+ssRNA
Incubation period, d	15‐45	30‐180	15‐160	30‐60	15‐60
Genome length, nt	7500	3200	9600	1700	7200
Envelope	No/quasi enveloped	Yes	Yes	Yes	No/quasi enveloped
Transmission	Fecal‐oral	Blood and other body fluids	Blood	Blood and other body fluids	Fecal‐oral
Infection course	Acute	Acute; chronic	Acute; chronic	Acute; chronic	Acute; chronic
Severity of hepatitis	±	++	+	+	±
Liver cancer development	No	Yes	Yes	Yes	Not clear
Vaccine	Yes	Yes	No	No	Yes (in China only)
Treatment	N/A	Yes	Yes	No approved medication^a^	No approved medication^a^

Abbreviations: ds, double‐stranded; FDA, Food and Drug Administration; N/A, not applicable; nt, nucleotide; ss, single‐stranded.

a For HDV, no FDA approved medication is available. Peg‐IFN‐α is the only recommended therapy, but the efficacy is unsatisfactory. For HEV, no FDA approved medication is available. Ribavirin has been used as off‐label treatment with good efficacy.

Host cells rapidly respond to viral infection by orchestrating a variety of cellular machineries. In particular, the mitochondrial compartment appears important in this respect and responds in various ways, including by acting as scaffold on which several key antiviral molecular machineries are converged.^4^ Mitochondria antiviral‐signaling protein (MAVS) acts as an adaptor for transcription and production of interferons (IFN), the most potent antiviral cytokines, in response to viral infection. Interestingly, different hepatitis viruses differentially interact with MAVS, resulting in enhancement or antagonism of host antiviral defense.^5^ In parallel, mitochondrial DNA (mtDNA) is able to elicit innate immune response through Toll‐like receptor 9 (TLR9) and stimulator of interferon genes (STING) signaling.^6^ Finally, the release of citric acid cycle intermediates from the mitochondrial matrix into the cytosol following viral infection also regulates host innate immunity.^7^ Together, these mechanisms likely impact on the infection course, pathogenesis, and the clinical outcome of IFN‐α treatment in hepatitis virus infections.

The liver is a metabolic powerhouse, and accordingly hepatocytes contain abundant numbers of mitochondria to support the energy requirement associated with high metabolic activity.^8^ Viruses require energy and macromolecule building blocks from the host to complete their life cycle but on the other hand can modulate the host metabolic machineries.^9^ Hepatitis viruses are known to regulate the number, quality, and dynamics of mitochondria, resulting in altered mitochondrial morphology and function.^10^ Accordingly, morphological and functional alterations of mitochondria are commonly observed in liver tissues obtained from viral hepatitis patients.^11^, ^12^, ^13^

Intriguingly, accumulating evidences have suggest that mitochondrial products serve as mediators of many cellular signaling pathways, including inflammatory responses that are prominent features of viral hepatitis. Adenosine 5'‐triphosphate (ATP), the primary carrier of energy, plays pleiotropic roles in inflammation by acting as an extracellular signaling molecule.^14^, ^15^ HCV replication actively consumes intracellular ATP.^16^ 5‐Aminoimidazole‐4‐carboxamide ribonucleotide (AICAR), an activator of ATP production, counteracts both HCV and HEV infection.^17^, ^18^ HBV infection decrease ATP levels in hepatocytes.^19^ Several other metabolites from mitochondria, in particular, citrate and succinate, are implicated in the pathological processes of viral hepatitis and cirrhosis.^20^, ^21^ Given the complexity, whether it is a sequential or causal relationship between mitochondrial alteration and hepatitis remains unclear.

## 2 MITOCHONDRIAL DYSFUNCTION IN VIRAL HEPATITIS PATIENTS

Mitochondrial dysfunction is associated with many common disorders.^22^ It is a prominent feature of liver cell injury and is often seen in patients with viral hepatitis. HBV and HCV infections are frequently accompanied by mitochondrial dysfunction. In patients, HCV infection results in morphological alteration of mitochondria, reduction in the copy number, and oxidative‐damage–triggered mutations in the genome of mtDNA.^11^, ^12^, ^13^, ^23^ Interestingly, mitochondrial abnormalities in HCV patients vary in a genotype‐dependent manner. Their frequency is higher in genotype 1b than genotype 2a/c or 3a infection, suggesting a greater intrinsic cytopathic effect of genotype 1b HCV.^11^, ^24^ The current direct‐acting antivirals are highly effective in inhibiting HCV infection. However, whether mitochondrial dysfunction persists in patients after HCV eradication remains an interesting question to be investigated. In HBV patients, a lower level of serum mtDNA content is related to an increased risk of HCC development, indicating that circulating mtDNA may be a potential noninvasive marker of HCC risk.^25^ Extensive mitochondrial gene dysregulation and global downregulation of mitochondrial function have been observed in HBV‐specific CD8 T cells from patients with chronic infection. Treatment with mitochondria‐targeted antioxidants restores antiviral activity of these exhausted HBV‐specific CD8 T cells.^26^ Data regarding the mitochondrial status in hepatitis A and E patients remain limited, identifying a need for future research.

## 3 THE MUTUAL INTERACTIONS BETWEEN HEPATITIS VIRUSES AND MITOCHONDRIAL COMPARTMENTS

### 3.1 Apoptosis in the pathogenesis of viral hepatitis

Accumulating evidence supports the role of liver cell apoptosis in the pathogenesis of viral hepatitis.^27^ Although there are multiple modes of programmed cell death, pyroptosis and apoptosis cascades through the extrinsic and intrinsic pathways are the predominant forms for viral hepatitis.^28^ The extrinsic signaling is activated via the cell surface death receptors including TNFR1, TRAIL‐R1, and Fas. The intrinsic pathway is mainly triggered by nonreceptor stimuli but characterized by the permeabilization of the outer mitochondrial membrane. This leads to the release of proapoptotic factors from the mitochondrial intermembrane space into the cytosol.^29^ A recent study demonstrates that the extrinsic and intrinsic apoptotic pathways activate pannexin‐1 to drive NLRP3 inflammasome assembly, which is involved in the pathogenesis of viral hepatitis.^30^, ^31^

The numbers of apoptotic hepatocytes in chronic hepatitis B and C patients are small but higher than those in healthy individuals.^32^ It is now generally accepted that cytotoxic T lymphocytes mediate the immune clearance of hepatitis virus‐infected hepatocytes. Immune‐mediated apoptosis plays an important role in liver damage and pathogenesis.^33^ However, hepatitis viruses may also have direct effects on apoptosis. The role of the HBV X gene product (HBx) in hepatocyte apoptosis is multifaceted. Proapoptotic function of HBx has been reported in hepatocytes of transgenic mice,^34^ whereas it also blocks Fas‐induced apoptosis in liver cells.^35^ Similarly, HCV infection enhances susceptibility to Fas‐mediated apoptosis,^36^ whereas several HCV proteins (core, E1, E2, and NS proteins) inhibit TNF‐α‐mediated apoptosis.^37^ Recently, HEV has been reported to induce hepatocyte apoptosis via mitochondrial pathway in Mongolian gerbils.^38^ However, the underlining interaction between apoptosis and HEV infection remains largely obscure.

**Figure 1:**
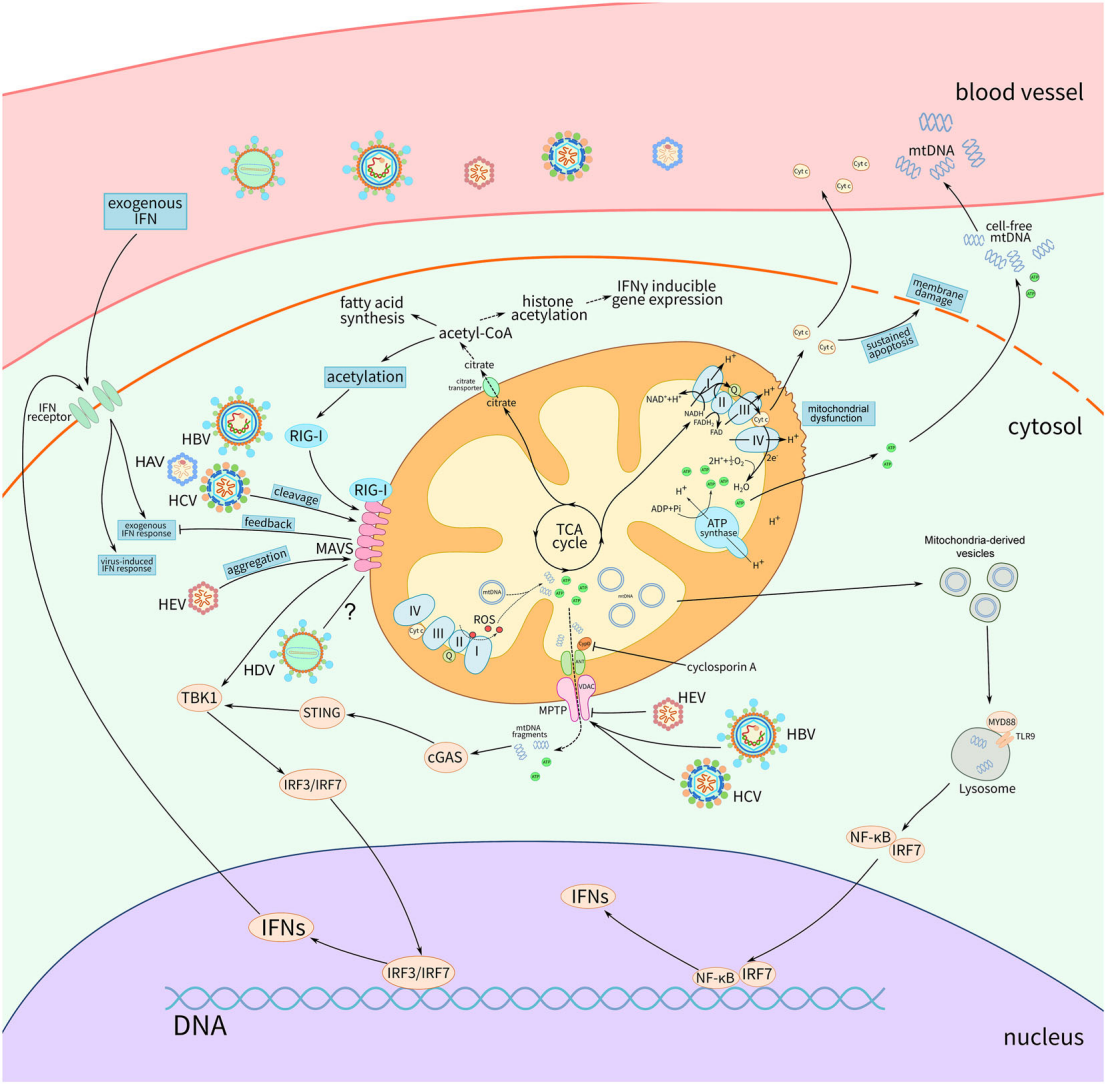
The mutual interactions of the mitochondrial compartment with hepatitis viruses and the consequences on the infections. Hepatitis viruses differentially modulate mitochondria antiviral‐signaling protein (MAVS) signaling. HAV and HCV cleave, while HEV induces MAVS aggregation. These interactions with MAVS result in enhancement or antagonism of innate immune response. Hepatitis viruses either induce or block the mitochondrial permeability transition pore (MPTP) opening, regulating the release of mitochondrial contents such as mitochondrial DNA (mtDNA) fragment or adenosine 5'‐triphosphate (ATP), which then lead to antiviral defense. mtDNA that are not completely degraded are able to enter the endocytic pathway through mitochondria‐derived vesicles, which engage Toll‐like receptor 9 (TLR9) in lysosomes and lead to the activation of the NF‐κB signaling and interferons (IFN) production. Sustained apoptosis caused by hepatitis virus infection triggers damage of membrane integrity, resulting in the liberation of mitochondrial contents into the extracellular milieu

Cytochrome c, an essential component of the electron transport chain (ETC) transferring electrons from complex III to complex IV, plays a key role in the early events of mitochondria‐mediated apoptosis. Serum cytochrome c has been suggested as a potential new marker for fulminant hepatitis in patients.^39^ During apoptosis, cytochrome c is released from the mitochondrial intermembrane space to induce caspase activation. HCV can induce,^40^ whereas HEV can block the release of cytochrome c from mitochondria to cytosol (Figure 1).^41^ The possible correlation between the amount of serum cytochrome c and the severity of hepatitis should be further explored for potential diagnostic relevance. Besides cytochrome c, mutual interactions between caspase activation and viral infection have also been observed.^42^ Several viruses express proteins that could be cleaved by the caspase protease, resulting in inhibition of apoptosis.^43^, ^44^ For example, the HCV viral nonstructural protein 5A can be cleaved by activated caspase, which subsequently translocates to nucleus to enhance the transcription of several NF‐κB target genes to inhibit apoptosis.^45^ The protein from HEV ORF2 has different forms and could translocate to the cell nucleus.^46^ However, whether ORF2 protein is cleaved by the host protease and whether it regulates apoptotic pathway remain to be further studied. Taken together, apoptosis is likely an important mechanism in pathogenesis of viral hepatitis. Hepatitis viruses can modulate apoptotic pathways at various levels. Thus, detection and quantification of particular apoptosis‐related molecules may be explored as potential biomarkers for disease diagnosis in viral hepatitis patients.

### 3.2 MAVS and mtDNA‐mediated Innate Immune Response

The early and non‐specific detection of hepatitis viruses is generally through the recognition by pathogen‐associated molecular patterns (PAMP) as innate immunity sensors. This leads to the activation of downstream IFN signal pathway and subsequent production of the ultimate antiviral effectors, interferon‐stimulated genes (ISG).^47^, ^48^ MAVS, acting as an adaptor for transcription and production of IFN, shows specific interactions with different hepatitis viruses. HAV and HCV provoke a blockade in cell‐autonomous IFN production by inducing proteolytic release of a part of the extra‐mitochondrial domain of MAVS. This is clinically supported by the presence of cleaved MAVS in the liver biopsies of HCV—but not HBV‐infected patients.^49^, ^50^, ^51^ The HCV protease NS3/4A cleaves MAVS off the mitochondria,^52^ whereas HAV uses a stable, catalytically active polyprotein processing intermediate to target MAVS for proteolysis.^49^ Instead of directly provoking MAVS proteolysis, HEV induces MAVS to form “prion‐like” polymers, producing a type III IFN response (Figure 1). The sequestering of MAVS in morphologically altered mitochondria may explain the relatively poor response to IFN treatment in the clinical management of HEV compared with that in HCV‐infected patients.^5^ Thus, exploring drugs preventing aggregation of MAVS on the outer membrane of mitochondria could be potentially used as a combination with IFN to enhance the anti‐HEV efficacy. HBV infection is another case altogether, and investigation of liver biopsies from chronic HBV patients indicates the absence of activated innate immune response.^53^ Thus, HBV is likely invisible to pattern recognition receptors, and the role of MAVS may not be prominent.

Because mtDNA contains remnants of bacterial nucleic acid sequences and is methylated in a different way from nuclear DNA, it resembles non‐self DNA and is thus easily to be degraded after transferring to the cytosol, leading to the activation of innate immune system.^54^ mtDNA‐mediated immune activation involves TLR9 and cGAS‐STING signaling pathways, which contribute to the clearance of invading pathogens and provoke inflammasome activation, interleukin‐1 production, and pyroptosis.^55^, ^56^ Because of bidirectional transcription, mtDNA is capable of generating overlapped transcripts. These formed long double‐stranded RNA structures that engage in MDA5‐mediated antiviral signaling to trigger a type I IFN response.^57^ Clinically, IFN treatment in HCV patients significantly decreases the frequency of mtDNA mutations in hepatocytes and increases the mtDNA copy numbers in peripheral leukocytes.^12^, ^58^ Moreover, mtDNA was reported to mediate IFN response.^59^ Even though hepatocytes contain hundreds of copies of mtDNA, it is possible that the combination of mtDNA deletions and point mutations, together with mtDNA strand breaks by increased reactive oxygen species (ROS), could reach a threshold sufficient to induce mitochondrial dysfunction, contributing to the pathogenesis of viral hepatitis. Very recently, it has been reported that new mtDNA synthesis can activate the NLRP3 inflammasome.^60^ As described, activation of NLRP3 inflammasome is closely related to the pathogenesis of chronic liver diseases, including viral hepatitis.^31^

### 3.3 Mitochondrial morphodynamics in response to hepatitis virus infection

**Figure 2:**
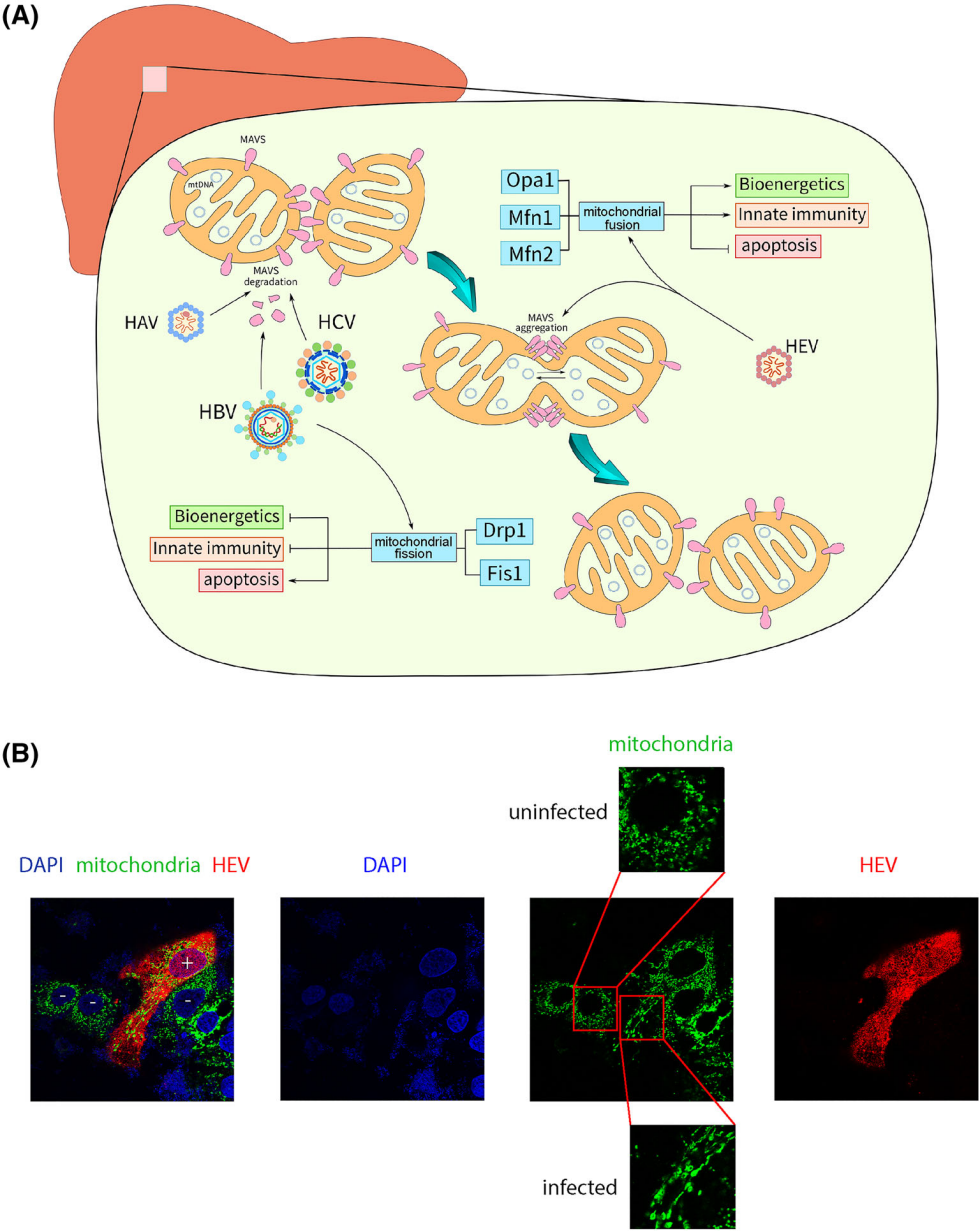
Mitochondrial morphodynamics is differentially regulated by hepatitis viruses to modulate innate immune response. A, The mitochondrial life cycle entails frequent fusion and fission events. Mitofusin‐1 (Mfn1), mitofusin‐2 (Mfn2), and optic atrophy 1 (Opa1) are the key regulators of fusion, whereas dynamin‐related protein 1 (Drp1) and mitochondrial fission 1 protein (Fis1) modulate fission. HBV and HCV induce fission, whereas HEV triggers fusion. B, Immunofluorescence staining of human liver cells infected with HEV showing the induction of mitochondrial fusion. HEV capsid protein (red; anti‐ORF2), mitochondria (green; anti‐HSP60), and 4',6‐diamidino‐2‐phenylindole DAPI (blue). Cells were visualized with 63× oil immersion lens at identical settings

The mitochondrial life cycle entails frequent fusion (in which two mitochondria form a single organelle) and fission (the division of one mitochondrion into two daughter organelles) events.^61^ These two opposing processes collaboratively control the number and size of mitochondria and maintain cell homeostasis. Mitofusin‐1 (Mfn1), mitofusin‐2 (Mfn2), and optic atrophy 1 (Opa1) are the key regulators of fusion, whereas dynamin‐related protein 1 (Drp1) tightly modulates fission (Figure 2A). A main reason for continual mitochondrial fission and/or fusion is that it facilitates the degradation of damaged organelles by mitophagy, which is regulated by Parkin and Pink proteins. It promotes mitochondrial turnover and prevents accumulation of dysfunctional mitochondria. HCV and HBV infections promote mitophagy.^62^, ^63^ The role of mitophagy in other hepatitis viruses needs to be further studied.

Upon infection, hepatitis viruses rearrange the intracellular microenvironment, including the mitochondrial compartment.^64^ Mitochondrial fission has been frequently observed in HBV and HCV infections.^10^, ^65^ HCV promotes fission by inducing Drp1 phosphorylation.^66^ This correlates with oxidative stress, presenting as excessive lipid peroxidation and deficiency of tissue hepatocellular antioxidant stores, which in turn contributes to steatosis that is highly prevalent in HCV infection.^67^, ^68^ In contrast, HEV is able to trigger mitochondrial fusion to promote viral replication (Figure 2B).^69^ Because mitochondrial fission is the initial step of mitophagy, the differential regulation of mitochondrial morphodynamics by HEV compared with HCV may suggest a negative regulation of mitophagy during its propagation.

The fission and fusion processes in hepatocytes are responsible for the exchange and reallocation of mitochondrial contents including mtDNA. Inhibition of mitochondrial fusion is related to mtDNA depletion.^70^ Importantly, the equilibrium between fission and fusion is crucial for stabilizing mtDNA copy number and maintaining healthy liver function.^71^ Hence, modulation of mitochondrial morphodynamics could potentially affect virus‐induced liver dysfunction.

In addition, morphodynamics also regulates innate immunity by affecting the distribution of MAVS on the mitochondrial outer membrane. As reorganization of MAVS spatial distribution is a key event in IFN production in response to viral infection, such spatial reorganization has important consequences. Mitochondrial fusion promotes, whereas fission inhibits, RIG‐I‐like receptor (RLR) signaling. Fibroblasts lacking mitofusin proteins produce less IFN and pro‐inflammatory cytokines upon viral infection.^72^, ^73^ Small molecules, such as mitochondrial division inhibitor 1 (Mdivi1) that inhibits Drp1 activity, have been developed.^74^ Hence, the effects of these agents on different hepatitis viruses are interesting be investigated.

### 3.4 The role of the mitochondrial electron transport chain

Mitochondrial ETC consists of a series of complexes that transfer electrons from donors to acceptors via redox coupled with the transfer of protons across a membrane. It is the site for oxidative phosphorylation and generation of ATP. Mitochondrial morphodynamics can regulate the respiratory rate.^75^ Fused mitochondria enhance, whereas mitochondrial fission decrease, respiratory function. Thus, changing the dynamics of mitochondrial fission and fusion influences mitochondrial function and constitutes an evident target for viruses to corrupt mitochondria‐mediated innate immunity. Hepatitis viruses actively interact with the ETC; for example, HBx protein down‐regulates ETC activity.^76^ HCV replication inhibits ETC and so the production of ATP.^77^ By profiling the role of different ETC complexes, complex III was found to support HEV replication.^18^

During cellular respiration, byproducts like ROS are produced under stressed conditions.^78^ Increased ROS production is associated with liver injury and the pathogenesis of viral hepatitis.^79^ Furthermore, ROS production is involved in various cellular signaling pathways, including those mediating immune responses. ROS can induce aggregation of MAVS on mitochondrial outer membrane to initiate IFN response. Cells with reduced ETC activity are impaired with production of IFNs and proinflammatory cytokines during viral infection.^80^ In contrast, increased ROS production counteracts HCV replication.^81^ Thus, the ETC emerges as a primary target for viral infection, although hepatitis viruses likely target its functionality indirectly, for instance, by modifying mitochondrial morphodynamics.

### 3.5 Mitochondrial permeability transition pore and hepatitis viruses

Mitochondria actively communicate with the cytosol and nuclear compartments. The signals involved are mediated through proteins located on the mitochondrial membrane, including the mitochondrial permeability transition pore (MPTP). Mitochondrial contents can escape from the mitochondrial matrix during MPTP opening.^82^, ^83^ The products related to the action of ETC, such as ATP and cytochrome c, are transferred through MPTP to cytosol to exert biological functions. MPTP is composed of voltage‐dependent anion channel (VDAC) in the outer mitochondrial membrane, the adenine nucleotide translocator (ANT) in the inner mitochondrial membrane, and cyclophilin D (CypD) as its regulator in the matrix.

Hepatitis viruses have various interactions with MPTP. HBx protein has been shown to colocalize with VDAC, leading to alteration of mitochondrial transmembrane potential. The 68‐117 region of HBx interacts with mitochondria and is necessary for membrane permeabilization.^84^ HEV ORF3 protein sustains high levels of oligomeric VDAC to preserve mitochondrial potential and membrane integrity, thereby protecting infected cells from mitochondrial depolarization and death.^41^ HBV and HCV core proteins provoke MPTP opening, whereas HEV prevents such an event. In line with this, the MPTP inhibitor cyclosporine A (CsA) inhibits HBV and HCV^85^, ^86^, ^87^ but promotes HEV replication.^18^, ^88^ As highlighted, the importance of mtDNA in innate immunity, mtDNA fragments in fact are also released through MPTP. Thus, targeting MPTP opening represents a potential antiviral strategy.

## 4 THE IMPACT OF MITOCHONDRIAL METABOLITES

Metabolites produced from the mitochondrial tricarboxylic acid cycle, including citrate, succinate, fumarate, and acetyl‐CoA, are important regulators of signaling transduction when released from the mitochondria.^56^, ^89^ Citrate synthase and succinate dehydrogenase are up‐regulated in HBV‐infected cells, leading to elevation of the corresponding metabolites such as fumarate and succinate.^90^ Succinate has been recognized as an emerging signal transducer to activate inflammatory pathways.^7^ An example is the increase in antigen‐presenting capacity of dendritic cells if cytosolic succinate levels increase.^91^ Thus, it is rational to suggest that such molecules may modulate innate immunity in hepatocytes as well.^92^ HCV infection has been related to elevated level of acetyl‐CoA, a metabolite that participates in many biochemical reactions in protein, carbohydrate, and lipid metabolism.^93^ It has been widely recognized that acetyl‐CoA contributes to lysine acetylation by donating its acetyl group.^94^ Lysine modification controls many aspects of protein function and provides an obvious mechanism as to how acetyl‐CoA can influence cellular function. HBV replication is regulated by the acetylation status of the cccDNA‐bound H3/H4 histones.^95^, ^96^ Acetylation of retinoic acid‐inducible gene I (RIG‐I) regulates its antiviral functions,^97^ and RIG‐I is essential for sensing HAV,^98^ HBV,^99^ HCV,^100^ and HEV infections.^101^ Importantly, adequate cytosolic acetyl‐CoA level is required for interferon‐γ (IFNγ) production.^102^ Other metabolites can inhibit inflammatory responses. For example, lactate acts through the lactate receptor to reduce hepatitis in mouse models.^103^ There is an increase in lactate production in HCV‐infected cells, probably because the corruption of mitochondrial function provokes increased dependency in the hepatocyte on glycolysis to support its energy needs.^104^ In apparent agreement, targeting mitochondrial metabolism has been proposed to prevent chronic neuroinflammation.^105^ This may bear implications for treating neurologic diseases caused by HEV infection.^106^

## 5 IMPLICATIONS FOR THERAPEUTIC DEVELOPMENT

IFN‐α has been used in the clinic for decades to treat chronic HBV and HCV infections. The effects of IFN on viral replication have been linked to mitochondrial functions,^107^ but conversely, mitochondria regulate antiviral IFN responses via MAVS or the production of ROS. The development of direct‐acting antivirals (DAA), in particular, the nucleoside/nucleotide analogues, constitutes a landmark in advancing the treatment for viral hepatitis.^108^ Nucleoside/nucleotide analogues can efficiently inhibit viral replication by inhibition of viral polymerase activity.^109^ However, these drugs may exert off‐target effects by inhibition of mitochondrial DNA polymerase, resulting in a reduction of mtDNA copy number, although a minor reduction may not present a clinically apparent phenotype.^110^, ^111^ Fialuridine, a nucleoside analogue investigated for treating HBV infection, caused five deaths from liver failure associated with lactic acidosis and two required liver transplantation.^112^ The toxicity is primary due to damaging mitochondria, particularly in nerves, liver, skeletal, and cardiac muscle, as these tissues contain many mitochondria.^113^ The degree of these side‐effects limits development of this class of drugs, even though the antiviral effect may be very promising.

Despite the launch of many antiviral drugs, new therapeutics are still required for eliminating viral hepatitis. Unlike HCV, the persistence of cccDNA prevents cure but only inhibits viral replication in HBV patients.^114^ For HEV, besides supportive care and off‐label treatment with ribavirin or IFN‐α for some cases, there is no proven antiviral medication available. Mitochondria represent a viable target for new therapeutic development. As mitochondrial dysfunction is widely present in HBV patients, treatment with mitochondria‐targeted antioxidants mitoquinone (MitoQ) and the piperidine‐nitroxide MitoTempo can restore the antiviral activity of HBV‐specific CD8 T cells.^26^ MitoQ is based on the delivery of a potent antioxidant with targeted lipophilic cations that leads to accumulation up to several‐hundred fold in mitochondria. It has been extensively studied and demonstrated safety in humans.^22^, ^115^, ^116^ Because increased oxidative stress and subsequent mitochondrial damage are the key mechanisms causing pathogenesis in viral hepatitis, treatment with MitoQ can decrease liver damage in HCV patients.^115^ It has also been shown to attenuate liver fibrosis in mice.^117^

The mitochondrial ETC complexes have long been recognized as an antiviral target.^118^ The complex I inhibitor, metformin, inhibits HBV and HCV infections in experimental models,^119^, ^120^ although the effects in patients remain unclear. Complex III sustains HEV replication and can be targeted by pharmacological inhibitors to inhibit viral replication in experimental models but requires further clinical validation.^18^

Lastly, mitochondria‐mediated apoptosis is essential in the pathogenesis of viral hepatitis; however, no optimal drug has been identified to prevent or treat liver injury. In this respect, mitochondria‐targeted antioxidants or caspase inhibitors, look promising, but require further investigation.

## 6 CONCLUDING REMARKS

Liver cells are enriched in mitochondria that support the unique features of hepatic metabolism but also orchestrate cell‐autonomous antiviral immunity upon viral infection. Mitochondrial dysfunction commonly occurs in viral hepatitis patients. This associates with disease progression from acute, chronic infection to cancer development. Hepatitis viruses actively interact with the mitochondrial compartment at various levels, including regulation of mitochondrial morphodynamics, innate immune response, bioenergetics, and metabolism. The mode of actions of these interactions may differ among the five major types of hepatitis viruses but are essential for understanding the pathogenesis, clinical outcome, and treatment response in viral hepatitis patients.

The prominent role of mitochondria in contributing to pathology has provided opportunities for therapeutic development against viral hepatitis and prevention of liver cancer development. Several mitochondrial‐related or targeted agents have been used in the clinic or tested in clinical trials, including the complex I inhibitor metformin, the MPTP inhibitor CsA, the NAD^+^ precursor nicotinamide mononucleotide, the mitochondria‐targeted protective compounds MitoQ and Bendavia, and the antioxidant coenzyme Q_10_. However, the development and application of mitochondria‐related therapies remain at their infancy. We propose to enhance the therapeutic development by identifying and repurposing the existing FDA‐approved medications with mitochondria‐targeted properties. On the other hand, dietary and herbal supplements^121^ and other new approaches^122^, ^123^ should also be explored for their potential to modulate or restore mitochondrial function.

## CONFLICT OF INTEREST

The authors declare that they have no conflict of interest.
